# Retroperitoneal haemangioma masquerading as recurrence of well-differentiated neuroendocrine tumour: a cautionary note

**DOI:** 10.1308/rcsann.2025.0009

**Published:** 2025-03-25

**Authors:** AM AbdelDayem, S Navalkissoor, TV Luong, M Caplin, R Mirnezami

**Affiliations:** ^1^ Royal Free London NHS Foundation Trust, UK; ^2^ Cairo University, Egypt; ^3^ ENETS Centre of Excellence, UK

**Keywords:** Retroperitoneal haemangioma, Neuroendocrine tumour, ^68^Ga-DOTATATE PET/CT, Gallium-avid

## Abstract

Gallium 68-DOTATATE positron emission tomography scanning is the cornerstone of nuclear medicine imaging for neuroendocrine tumours. Previous reports have demonstrated the potential for vertebral haemangiomata to mimic neuroendocrine skeletal metastases. We present the case of a 42-year-old man with a history of well-differentiated neuroendocrine tumour of the appendix, identified incidentally following emergency appendicectomy. Postoperative ^68^Ga-DOTATATE positron emission tomography/computed tomography scanning revealed an intensely gallium-avid retroperitoneal lesion adjacent to the right psoas muscle. Surgical excision of this lesion was recommended following multidisciplinary team discussion. Histological evaluation of the resected lesion revealed an intravascular capillary haemangioma. Haemangiomas can mimic residual or recurrent neuroendocrine tumour based on gallium avidity, and this should be considered in atypical cases.

## Background

Neuroendocrine tumours (NETs) of the appendix are rare neoplasms, often discovered incidentally following appendicectomy for suspected acute appendicitis.^1^ Typically well differentiated, these tumours generally have a favourable prognosis and according to current guidelines, patients with tumours <1cm in size, completely excised (R0) and exhibiting no high-risk histological features require no surveillance. The North American Neuroendocrine Tumor Society and European Neuroendocrine Tumour Society guidelines describe the specific indications for surveillance imaging in the context of appendiceal NETs.^2^

Somatostatin receptor (SSR) imaging, e.g. gallium 68-DOTATATE positron emission tomography/computed tomography (^68^Ga-DOTATATE PET/CT), studies are commonly used for the detection of metastatic or recurrent disease in patients with well-differentiated NETs owing to their high sensitivity and specificity for SSR-positive tissues.^3^

In the literature, other benign and malignant conditions can express SSR including vertebral haemangiomas, which have been reported to demonstrate increased uptake on ^68^Ga-DOTATATE PET/CT imaging, perhaps because of their rich vascularity, their expression of SSRs or high capillary permeability.^4–8^ This can theoretically lead to false-positive findings in patients undergoing surveillance for NETs, because the increased tracer accumulation mimics metastatic or recurrent disease. In this case report, we describe such a situation involving a 42-year-old man with a history of appendiceal NET.

## Case history

The 42-year-old man in this case study underwent a laparoscopic appendicectomy in February 2023 in Uganda for perforated appendicitis. Histopathological assessment revealed a fully resected (R0) 4mm well-differentiated NET of the appendiceal tip. Final staging confirmed this as a pT2 tumour with no evidence of lymphovascular or perineural invasion, with a Ki-67 proliferation index of <1%. The postoperative course was complicated by intra-abdominal collections for which he underwent a laparotomy and washout. We are not aware of any further CT imaging performed prior to emergency laparotomy and washout, and this appears to have been undertaken on clinical grounds alone.

About one year later, the patient presented to our hospital having experienced lower abdominal discomfort a few weeks prior. Physical examination found him to be generally well, with a healed midline incision and healed laparoscopic ports sites. Because of his history, he underwent a CT enterography.

**Figure 1:**
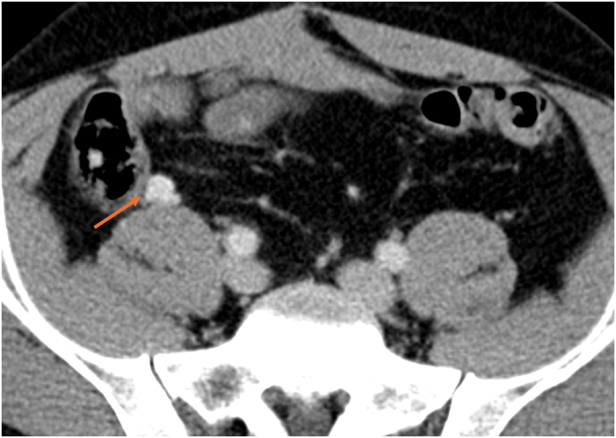
A 13 × 11mm hypervascular lesion is seen anterior to the right psoas muscle on portovenous phase imaging (arrow)

**Figure 2:**
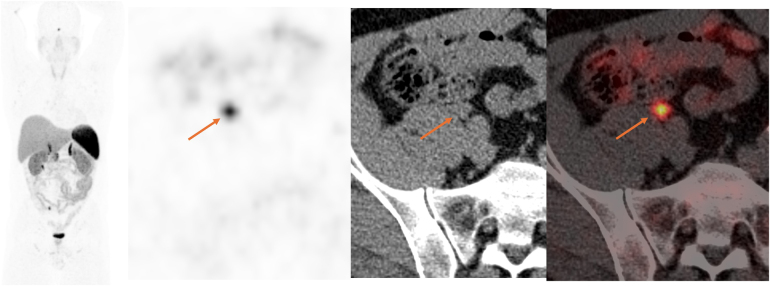
68Ga-DOTATATE maximal intensity image, positron emission tomography (PET), computed tomography (CT) and PET/CT demonstrates intense uptake in the lesion anterior to the right psoas muscle (arrows), Krenning score 3.

The scan revealed an enhancing 13 × 11mm nodule anterior to the medial border of the right psoas muscle, which prompted a referral to the neuroendocrine tumour unit at The Royal Free Hospital (London, UK). A ^68^Ga-DOTATATE PET/CT scan was undertaken and this demonstrated a single intensely avid lesion at this location. On the basis of the intense avidity, and given the patient’s previous history, this was interpreted as being potentially in keeping with an area of residual or recurrent/metastatic neuroendocrine tumour (Figures 1 and 2).

The case was discussed at the Royal Free Hospital neuroendocrine tumour multidisciplinary team meeting, and surgery was recommended to excise the lesion. Surgery involved a laparotomy, extensive adhesiolysis, mobilisation of the right colon and terminal ileum, isolation of the right ureter and gonadal vessels and excision of a single soft tissue nodule corresponding to the preoperative imaging. No other abnormalities were identified at laparotomy.

**Figure 3:**
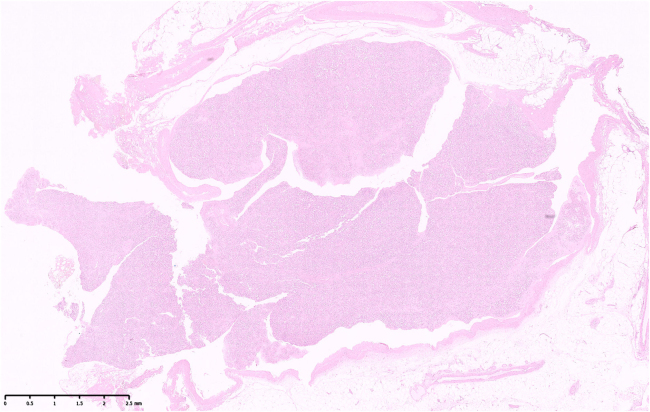
Low magnification hematoxylin and eosin (H&E) staining of the entire lesion showing an intravascular mass, entirely intraluminal, obstructing the lumen

**Figure 4:**
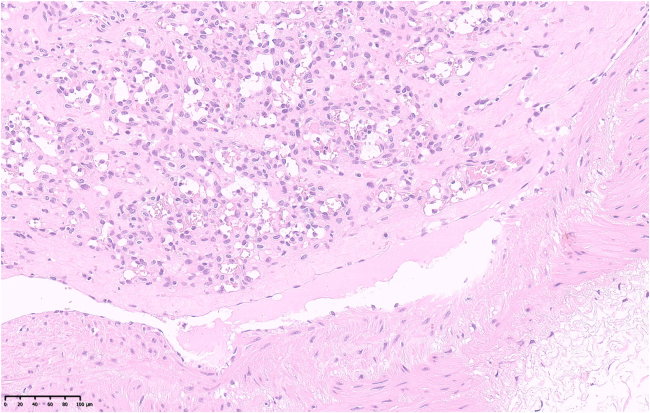
High-magnification H&E-stained section of the tumour, seen to consist of lobular proliferation of capillaries. No atypia, mitotic activity or necrosis is seen.

**Figure 5:**
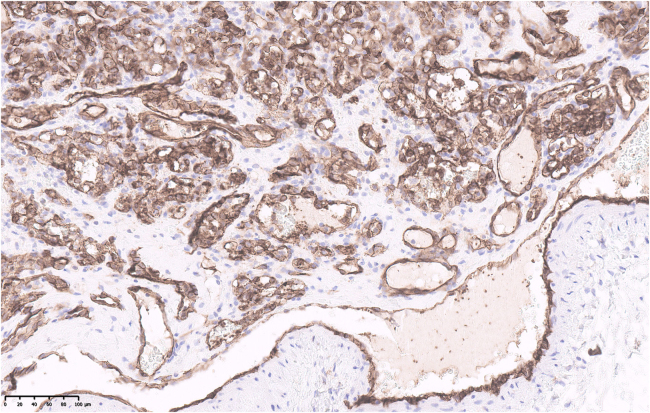
Immunohistochemical staining confirming that the intraluminal mass is positive for CD31 (endothelial marker) as well as the endothelial cells lining the vessel

Histopathological examination of the excised lesion revealed features in keeping with an intravascular capillary haemangioma (Figures 3–5).

There was no evidence of residual or recurrent neuroendocrine tumour. The patient was discharged home on the sixth postoperative day after an uncomplicated recovery. Three months postoperatively, the follow-up PET/CT scan confirmed the disappearance of the para-psoas lesion identified on the earlier scan.

## Discussion

NETs of the appendix account for an estimated 50–77% of all appendiceal neoplasms. In most cases, these tumours are of low grade with a generally favourable prognosis. However, the risk of metastasis, particularly with large tumours, underscores the importance of vigilant follow-up.^9^ ^68^Ga-DOTATATE PET/CT imaging is a widely used modality in the surveillance of NETs owing to its high specificity for SSR-positive tumours. However, false-positive results can occur and there are few benign differential diagnoses for the accumulation of SSR analogues. ^68^Ga-DOTATATE can accumulate in benign inflammatory diseases, because activated macrophages and lymphocytes express SSRs on their surface.^10^ The exact mechanism of uptake in these benign lesions is not clear.

In this case, a 42-year-old patient with a history of appendiceal NET underwent imaging for abdominal pain, which identified an avid right retroperitoneal lesion, initially suspected to represent metastatic disease. However, histopathological examination revealed the lesion to be an intravascular capillary haemangioma, a benign vascular lesion. This finding highlights a significant cautionary point in the interpretation of ^68^Ga-DOTATATE PET/CT scans, where vascular lesions like haemangiomas may exhibit increased tracer uptake. Although rare, these false-positive results have been documented in vertebral haemangiomas and other benign conditions, creating diagnostic challenges in NET surveillance.

The sequence of events, starting from the resection of an appendiceal NET, and a suspicious right retroperitoneal lesion on follow-up CT, further shown to be intensely avid on ^68^Ga-DOTATATE PET/CT, raised concern for residual or recurrent NET; hence the decision to proceed with resection in this patient, following multidisciplinary discussion. Subsequent follow-up imaging confirmed resolution of the previously noted para-psoas lesion.

## Conclusions

Haemangiomas can display increased uptake on ^68^Ga-DOTATATE PET/CT scans leading to confusing diagnoses in known NET patients. Although these scans are highly sensitive for detecting SSR-positive tissues, false positives can occur in benign conditions, such as haemangiomas, owing to their vascular nature or potential SSR expression. Our report demonstrates that caution is needed when interpreting SSR-positive foci, particularly if the contrast-enhanced cross-sectional imaging is not typical of metastatic disease. In unusual cases in which there is diagnostic uncertainty, serum measurement of ‘chromogranin A’ levels may further assist in the diagnostic work-up of patients being considered for surgery.
